# Introductory to the Fifteenth Volume

**Published:** 1859-06

**Authors:** 


### EDITORIAL DEPARTMENT.

*Introductory to the Fifteenth Volume.*—The present number of our Journal, though the same in its arrangement as it has been for thirteen years, it having been issued in a much smaller form during the first year of its existence, presents a change upon its title page which demands an explanation to those who have known it so long in its late form. Circumstances have determined us to make the city of New York our permanent residence instead of Buffalo; and being loth to lose an occupation which we have enjoyed so much, and upon which we had expected to bestow a good part of the labor of a life-time, and unwilling to see a work which was projected and carried on for ten years by our father before us pass out of our hands, we have determined upon issuing the Buffalo Medical Journal with the modification which appears upon the cover, simultaneously in the cities of New York and Buffalo.

In taking this important step, we weighed the matter as carefully in regard to the future of the Journal, as in the case of our personal interests. It is unnecessary for us to mention how well our profession is supported in the great metropolis by its periodical medical literature; and we can only say that it is farthest from our thoughts to consider that the able and eminent men who conduct and support the venerable "New York Journal of Medicine," the "American Medical Monthly," the "American Medical Gazette," and the "Medical Press" have left a void in that department; and while we confidently expect to command a larger amount of valuable practical matter after our removal to New York, we shall still retain our position as the Journal of Western New York, losing none of our former advantages, and gaining, we hope, in our sources of information.

In our original department, we shall be enabled to enlist writers of distinction whose names have not been often before our readers; and we expect in a few months to publish a series of physiological articles, investigating some of the subjects in this branch upon which so much labor is being bestowed by the experimental physiologists of the old world, in the manner which is making rapid progress in our own country. We shall also retain the aid of our old Buffalo contributors, and our readers will often see the name of the first editor of this journal, Professor Austin Flint, who also makes the city of New York his residence, and will be a frequent contributor to our pages. This department will also contain regular reports from the Academy of Medicine and the Pathological Society, as well as occasional matter from other learned bodies, and clinical lectures. These reports we shall be enabled to make in most instances ourselves, and intend to use our best judgment and industry to render them interesting and instructive ; regarding this as one of the most important of our editorial labors. Our eclectic department will as usual contain selections of interesting matter from our exchange journals ; and in addition to this, we shall introduce into our editorial department numerous condensed abstracts of articles containing original ideas published in American and foreign journals : in this way, by perfecting our exchange list, and subscribing to foreign periodicals of value which we are not able otherwise to obtain, we shall be able to present in a small space, what is new in the medical world. Our editorial experience leads us to believe that we can be of more use to the profession by making carefully selected reports from the proceedings of societies, and well-digested abstracts of original articles which are not likely to come under the eye of our readers, than by editorials upon ephemeral subjects which agitate for the time the restless portion of the medical world. When great and important subjects are up for discussion, and a decided stand must be taken by the periodical-medical press, we will endeavor to sustain the right fearlessly and without hesitation ; but shall direct most of our labors to keeping our readers informed of the advancement of our science.

Finally, let us venture to hope that our old contributors will still continue to support us ; that our old subscribers will be satisfied and pleased with our change, and with our future efforts in their behalf ; and that our brethren of the press of New York will extend to us a cordial right hand of fellowship, and allow us the honor of working by their side in our common cause.

